# Pomegranate peel: Bioactivities as antimicrobial and cytotoxic agents

**DOI:** 10.1002/fsn3.3963

**Published:** 2024-03-05

**Authors:** Lubna F. Abu‐Niaaj, Hala I. Al‐Daghistani, Ibrahim Katampe, Bashaer Abu‐Irmaileh, Yasser K. Bustanji

**Affiliations:** ^1^ Department of Agricultural and Life Sciences, John W. Garland College of Engineering, Science, Technology, and Agriculture Central State University Wilberforce Ohio USA; ^2^ Department of Medical Laboratory Sciences, Faculty of Allied Medical Sciences Al‐Ahliyya Amman University Amman Jordan; ^3^ Hamdi Mango Center for Scientific Research The University of Jordan Amman Jordan; ^4^ College of Medicine University of Sharjah Sharjah United Arab Emirates; ^5^ Department of Pharmaceutical Sciences, Faculty of Pharmacy The University of Jordan Amman Jordan

**Keywords:** anticancer, antimicrobials, food spoilage, pomegranate peel

## Abstract

This is a comparative study to evaluate the effectiveness of six pomegranate peel extracts (PPEs) as antibacterial and antiproliferative agents. The Six PPEs were prepared using four solvent systems and each filtrate was concentrated to a gummy material to be used in the evaluation. The well‐diffusion method was used to evaluate their antimicrobial activity against bacteria typically associated with food spoilage: *Escherichia coli*, *Pseudomonas aeruginosa*, *Salmonella typhimurium*, *Listeria monocytogenes*, *Staphylococcus epidermidis*, *Staphylococcus aureus*, and three *Bacillus* species*.* The 3‐(4,5‐dimethylthiazol‐2‐yl)‐5‐(3‐carboxymethoxyphenyl)‐2‐(4‐sulfophenyl)‐2H‐tetrazolium (MTT) was used to evaluate the cytotoxicity against colorectal carcinoma cells (HCT116), prostate adenocarcinoma (PC3), ovarian cancer cells (SKOV‐3), and fibroblasts (MRC‐5). The antioxidant evaluation was done using the 2,2‐diphenyl‐1‐picrylhydrazyl‐hydrate (DPPH) assay. The pH of the water‐containing extracts was acidic and almost the same over 6 weeks. The six PPEs inhibited the bacterial growth in a comparable level to standard antibiotics. The effectiveness of each extract was dependent on the bacterial strain, and the *Listeria* showed a remarkable inhibition when exposed to the aqueous extract prepared at room temperature (RT). The aqueous (RT) and methanol PPEs had a significant antioxidant scavenging capability and a remarkable cytotoxic activity against the PC3 with half maximal inhibitory concentration (IC_50_) of 0.1 μg/mL. The boiled aqueous extract exhibited antiproliferative activity against HCT116 with an IC_50_ of 21.45 μg/mL. The effect on SKOV‐3 and fibroblasts was insignificant. With the exception of butanol, the antioxidant screening shows an inverse correlation between the polarity of the extraction solvent and the IC50 exhibited by the PPEs. The variation in the effectiveness of PPEs is suggested to be due to variable soluble bioactive compounds that may interact differently with different cells, though water‐containing extracts are promising antibacterial agents. The findings clearly show that pomegranate peel possessed the potential to be an eco‐friendly novel source for natural compounds that can be implemented in the food industry as a natural antimicrobial and natural food additive to prevent foodborne illnesses.

## Graphical abstract

The article is a comparative study to evaluate the effectiveness of six pomegranate peel extracts (PPEs) as antimicrobial agents against bacteria associated with food spoilage, and to evaluate their antioxidant and anticancer activities. The results will provide an insight for potential use of eco‐friendly extracts of pomegranate peel for industrial applications relevant to food spoilage and foodborne diseases. In addition, these bioactive extracts could be used as a natural source for food preservatives, antioxidants, and anticancer agents.

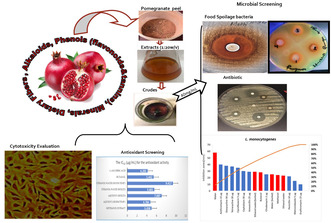

## 1 INTRODUCTION

Plants have evolved to produce various secondary metabolites as a defense mechanism against biotic and abiotic stresses. Such metabolites have chemically variable structures as they include alkaloids, tannins, vitamins, and phenolic compounds (Abu‐Niaaj & Katampe, 2018; Ekiert & Szopa, 2020; Mo et al., 2022; Wang, Alseekh, et al., 2019; Wang, Chen, et al., 2019). The plants' use in traditional medicine has been popular since ancient times because of their ability to boost the immune system and treat many illnesses. Hundreds of extracts and pure compounds of plants were reported to exhibit a wide range of bioactivities, including antimicrobial (Al‐Daghistani et al., 2021; Al‐Khresieh et al., 2022; Chen et al., 2020), anticancer (Alruwad et al., 2023), and antioxidant properties (Greenwell & Rahman, 2015). In addition, in vitro studies have proven their relaxation effect on vertebrates' smooth muscle in different organs (Abu‐Niaaj et al., 1993, 1996, 2019). It is documented that the natural compounds are often extracted from plants as a mixture which might cause a synergistic effect of some of their physiological activities (Melgarejo‐Sánchez et al., 2021). The content of bioactive compounds varies in different parts of plants; however, it is often high in peels that are usually discarded. Thus, there is an increased interest in developing innovative pathways for using plant peels in industrial applications, including cosmetics, animal feeds, and natural food preservatives and colorings.

Pomegranate (*Punica granatum* L. family Punicaceae) is a fruit that has a sweet taste and is revered for its valuable nutritional and health benefits. The variety of natural compounds in pomegranate includes minerals, alkaloids, and phenolic compounds such as flavonoids (anthocyanins and catechins) and tannins (ellagitannins and ellagic acid derivatives: punicalagin, punicalin, and pedunculagin) (Devanesan et al., 2018; Melgarejo‐Sánchez et al., 2021; Mo et al., 2022; Pirzadeh et al., 2021; Tamborlin et al., 2020). The content of such natural products is high in the pomegranate peel, which makes up to 50% of the fruit (Barathikannan et al., 2016). Different PPEs were reported to exhibit anti‐inflammatory, anticancer, and antimicrobial properties (Belgacem et al., 2021; Houston et al., 2017; Laurindo et al., 2022; Moradi et al., 2020; Rongai et al., 2017), and were also reported for their potential impact to treat heart diseases, blood pressure, obesity, diabetes, and to improve fertility in animals (Doostkam et al., 2020; El‐Sheshtawy et al., 2016; Govindappa, 2015; Hosseini et al., 2016). The displayed antibacterial activity was against a group of Gram‐positive and Gram‐negative bacteria including *Escherichia coli*, *Listeria monocytogenes* (Belgacem et al., 2020), *Pseudomonas aeruginosa*, *Staphylococcus aureus*, *Salmonella and Bacillus* spp., *Vibrio parahaemolyticus*, *Clostridia*, and *Yersinia enterocolitica* (Akhtar et al., 2015; Sorrenti et al., 2019; Wu et al., 2018). Despite the many extraction techniques used to highlight the importance of pomegranate peel as a source of bioactive compounds for industrial uses, collective findings are inconsistent due to the lack of standardized methodologies for extracting the most effective bioactive materials (Singh et al., 2023). This makes it difficult to assess the sufficient and safe use of pomegranate peel in industrial applications, especially those relevant to food preservations and pharmaceutical uses. Therefore, it is crucial to conduct a comparative study using different PPEs prepared by a standard protocol to evaluate diverse biological activities. To the authors' knowledge, our study is the first to compare the bioactivities of six PPEs extracted using solvents of different polarities and evaluating the effect of high temperatures on the activity of the water‐containing extracts. This study evaluates the effectiveness of PPEs as antimicrobial agents against bacteria associated with food spoilage and evaluates also their antioxidant and anticancer activities. The results will provide an insight for a potential eco‐friendly use of pomegranate peel for industrial applications relevant to food spoilage and foodborne diseases. In addition, results will indicate if these bioactive extracts could be used as a natural source for food preservatives, antioxidants, and anticancer agents.

## 2 MATERIALS AND METHODS

### 2.1 Preparation of pomegranate peel extracts

**FIGURE 1:**
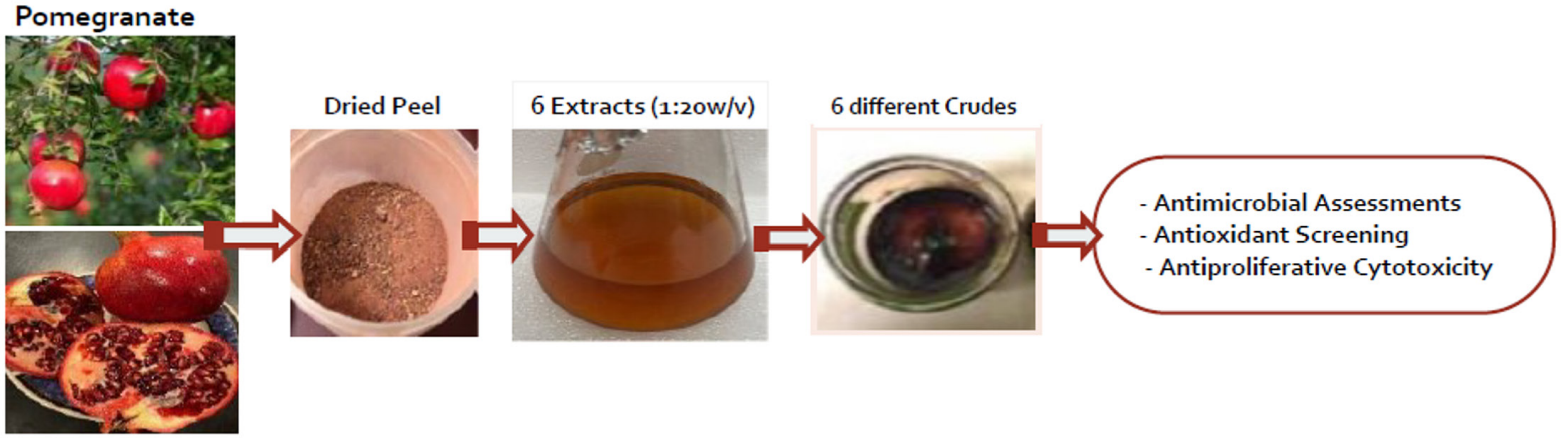
Preparation of the pomegranate peel extracts (PPEs) and crude.

The pomegranate, *Punica granatum* L., fruit was purchased from markets in the USA and washed before peeling. The dried peel at room temperature was pulverized in a blender and refrigerated until use. Six PPEs using four solvent systems were prepared: water, ethanol:water (1:1) mixture, methanol, and butanol. To obtain the extracts, 5 g of peel powder was macerated into 100 mL solvent (1:20 w/v) with stirring for 45 min at room temperature (RT). In addition, another set of the water and ethanol:water (1:1) extracts were prepared by boiling (bringing to simmer) for 45 min and then left to cool. The extracts were filtered through Whatman filter papers, and the filtrates were concentrated to a gummy material called the “crude” which was refrigerated until later use (Figure 1). A stock solution of 40 mg/mL of crude of each extract was prepared using sterile water. For the microbiology and cytotoxicity studies, dilutions were made from the stock solution upon need, and they were filtered through 0.45‐μm filters before use.

### 2.2 Measurement of pH

The stability of the water‐containing PPEs, without being concentrated, was evaluated by measuring the pH of the four water‐containing extracts: the aqueous and ethanol:water (1:1) mixture prepared at room temperature and by simmering. The daily pH was measured over 6 weeks using an UltraBasic Benchtop pH Meter (Denver Instrument, Denver, CO, USA).

### 2.3 Antibacterial screening

The antibacterial screening was carried out under sterile conditions following the guidelines of the Clinical and Laboratory Standards Institute (CLSI, 2020). The media and agar were prepared according to the manufacturer's instructions (Thermo Fisher Scientific, Waltham, MA, USA).

#### 2.3.1 Bacterial strains

The antibacterial activities of the six pomegranate extracts were assessed on a variety of Gram‐negative and Gram‐positive bacteria. The bacteria purchased from the American Type Culture Collection (ATCC) (Manassas, VA, USA) are listed in Table 1. The *Bacillus* species (*Bacillus subtilis*, *Bacillus megaterium*, and *Bacillus sphaericus*) were purchased from Carolina Biological (York Rd, Burlington, NC, USA).

**TABLE 1:** The list of the bacteria purchased from the American Type Culture Collection (ATCC).

Gram‐negative bacteria	Source	Gram‐positive bacteria	Source
*Salmonella typhimurium*	ATCC 103799	*Listeria monocytogenes*	ATCC 7644
*Escherichia coli*	ATCC 25922	*Staphylococcus epidermidis*	ATCC 12228
*Pseudomonas aeruginosa*	ATCC 27853	*Staphylococcus aureus*	ATCC 29213

For screening the antibacterial activity of the PPEs, the bacteria were inoculated in Mueller–Hinton broth and incubated in an incubator shaker at 30°C overnight. The cultures were then standardized at OD_600_ = 0.1 (contrast to 0.5 McFarland) to get 1.5 × 10^8^ CFU/mL before streaking onto the agar plates.

#### 2.3.2 Agar well‐diffusion method

Wells of 8 mm diameter were made into the Mueller–Hinton agar plates using a sterile borer. Each previously standardized culture (Section 2.3.1) was uniformly swabbed on the agar plates using sterile cotton applicators. Of each crude of PPE, 100 μL (40 mg/mL) was placed into a well, while the control well had distilled water. The plates were left aside until the liquid soaked into the agar before being incubated upside down overnight at 37°C. Then, the diameter of inhibition zones was measured in millimeters (mm). The screening of each PPE was done in triplicate against each bacterium and the reading was reported as an average of triplicate.

#### 2.3.3 Antibiotics susceptibility test

The antimicrobial activity was compared to the inhibition of selected antibiotics against the tested bacteria (Table 2). Using sterile cotton applicators, plates of Mueller–Hinton agar were streaked with a previously standardized bacterial inoculum (0.5 M McFarland equivalence) and were left to dry for a few minutes. The antibiotic disks were distributed on the agar surface, and plates were incubated at 37°C for 24 h before measuring the zones of inhibition in millimeters (mm), if any (CLSI, 2020).

#### 2.3.4 Minimum inhibitory concentration

The minimum inhibitory concentration (MIC) is the lowest concentration causing inhibition of the bacterial growth after 24 h. This study reported the MIC only against the Gram‐negative foodborne bacteria (*Salmonella typhimurium*, *Escherichia coli*, *Pseudomonas aeruginosa*). Using the stock solution of the crude of each PPE (40 mg/mL), five decreasing dilutions were made at twofold intervals (20, 10, 5, 2.5, and 1.25 mg/mL) with distilled water. The dilutions were filtered and 100 μL of each solution was placed in a well made into the agar plate, as described previously (Section 2.3.2). The plates were then incubated at 37°C overnight, and the inhibition zones were measured in millimeters (mm).

### 2.4 In vitro cytotoxicity screening using MTT assay

**FIGURE 2:**
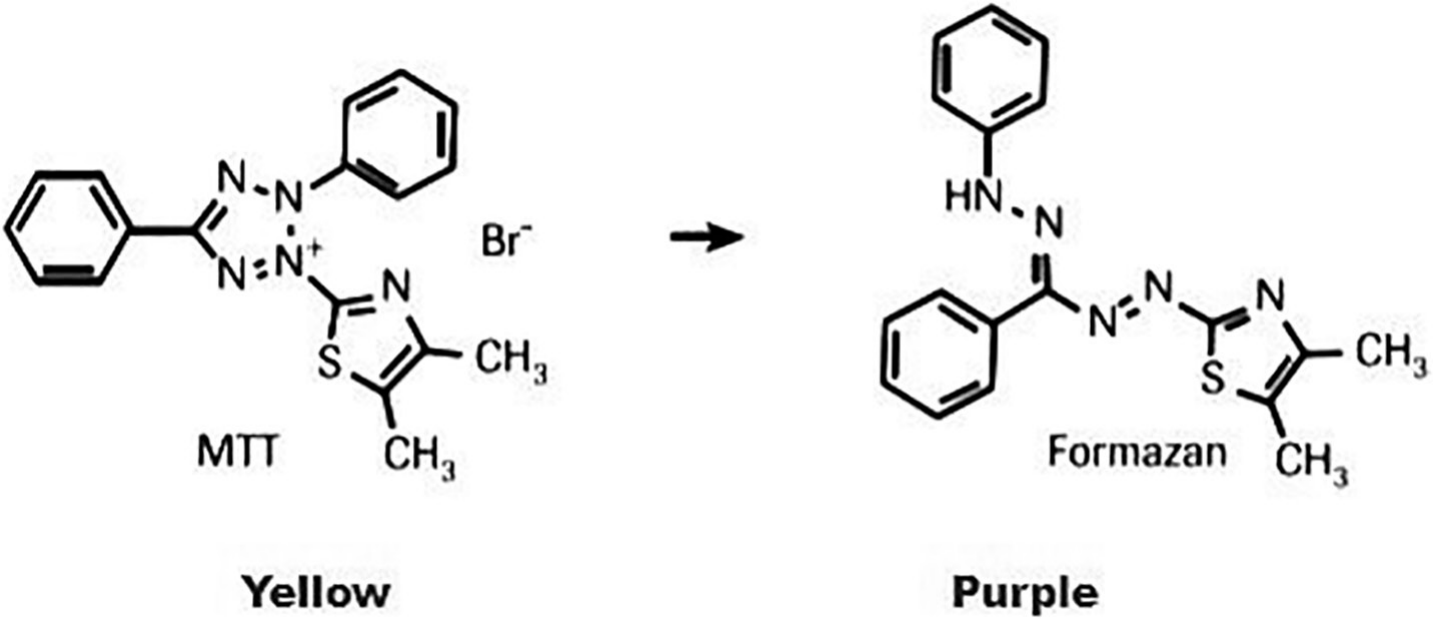
The enzymatic reduction of MTT (3‐(4,5‐dimethylthiazol‐2‐yl)‐5‐(3‐carboxymethoxyphenyl)‐2‐(4‐sulfophenyl)‐2H‐tetrazolium) to formazan by the mitochondrial reductase.

The effect of PPEs on the viability and proliferation of cancer cells and fibroblasts was assessed by measuring the cellular metabolic activity using a colorimetric assay. This assay uses the tetrazolium dye MTT (3‐(4,5‐dimethylthiazol‐2‐yl)‐5‐(3‐carboxymethoxyphenyl)‐2‐(4‐sulfophenyl)‐2H‐tetrazolium) which has a yellow color. A color change occurs due to the ability of the cellular oxidoreductase enzymes, which depends on nicotinamide adenine dinucleotide phosphate (NADPH) in viable cells, to reduce the yellow tetrazolium dye MTT producing purple crystals of insoluble formazan as given in Figure 2 (Kumar et al., 2018; Lü et al., 2012), with its optical density measured at λ 570 nm. The effectiveness of the PPEs on the proliferative activity of cells was evaluated based on criteria used to categorize the antiproliferative effect, according to the Geran et al. protocol (1972) adopted by the National Cancer Institute (NCI) plant screening program.

#### 2.4.1 Cell lines

The effect of PPEs on cell proliferation was evaluated against three lines of human cancer cells in addition to normal human fibroblasts. Two cancer cell lines were of the male and female reproductive systems, and the third cell line was of the digestive system. These cells were prostate adenocarcinoma PC3 (ATCC® CRL‐1435), ovarian cancer cell line SKOV‐3 (ATCC® HTB‐79), and colorectal carcinoma HCT 116 (ATCC® CCL‐247). The fibroblasts were MRC‐5 (ATCC® CCD‐1064Sk).

#### 2.4.2 Cell culture

The fibroblasts and cancer cell lines were cultured in different media. The fibroblasts were cultured in Iscove's Modified Dulbecco's medium (IMDM), while the cancer cells were cultured in Roswell Park Memorial Institute (RPMI) media (EuroClone, USA). Both media were supplemented with 10% fetal bovine serum (FBS), 1% of 2.0 mM l‐glutamine, penicillin (100 U/mL), and streptomycin (100 μg/mL) (EuroClone, USA). The cells were grown in 75 cm^2^ culture flasks (Membrane Solutions®, USA) and maintained at 37°C in a NuAire incubator supplemented with 5% carbon dioxide (CO_2_) (NuAire, USA).

#### 2.4.3 MTT cell viability assay

Cells were placed in 96‐well culture plates with 100 μL of media per well and were allowed to adhere by incubating the plates overnight at 37°C in the NuAire incubator supplemented with 5% CO_2_ in a humid environment. After reaching 80% confluence, cells were treated with six half‐fold diluted concentrations of the crude (100, 50, 25, 12.5, 6.25, and 3 μg/mL). The wells of control cells had sterile water in a volume equivalent to PPEs added to cells in treated wells. The plates were kept in the designated incubator for 72 h. The MTT assay was conducted using the CellTiter 96󠅣 Non‐Radioactive Cell Proliferation Assay Kit® (Promega, USA). The inhibition of cellular proliferation is a percentage of absorption of treated cells relative to that of the control (untreated cells) at λ 570 nm. The absorption was measured using a microtiter plate reader (BioTek, USA) 72 h posttreatment. The experiments were done in triplicate, and the mean was calculated and used to determine the half maximal inhibitory concentration (IC_50_) which represents a 50% decrease in viability of cells.

### 2.5 Antioxidant activity using l‐ascorbic acid as a standard

The DPPH assay is a low‐cost and quick method widely employed to evaluate the antioxidant activity of natural products. The DPPH solution absorbs strongly at λ 517 nm due to its deep violet color resulting from its unique electron. When a free radical scavenger is present, the electron pairs up, leading to a loss of absorption and a corresponding reduction in the number of acquired electrons. The change in absorption is used to determine the antioxidant potential of the tested sample. This study evaluated the scavenging effect of the DPPH radical by utilizing l‐ascorbic acid as a reference standard, following established protocols with adjustments to accommodate the use of 96‐well plates (Baliyan et al., 2022; Boly et al., 2016; Sanchez‐Moreno et al., 1998). Briefly, a DPPH reagent was freshly prepared by dissolving 2 mg in 51 mL methanol (HPLC (high performance liquid chromatography) grade, Fischer, USA). In a 96‐well plate, 150 μL DPPH reagent was added to wells containing 5 μL of the desired PPEs (*n* = 3) and the plate was incubated for 30 min at room temperature in the dark before measuring the absorption at λ 517 nm. The crude of each PPE was diluted to match the same in‐well concentrations as the l‐ascorbic acid, as described later in this section. The average of each triplicate was recorded and the percentage of inhibition was calculated according to Equation (1).

(1) $$\begin{array}{c} \%\text{Inhibition}=\\ \;\frac{\left(\text{Average Absorbance of blank}‐\text{Average absorbance of sample}\right)*100}{\text{Average absorbance of blank}} \end{array}$$

The blank well had only the DPPH. The standard curve of the l‐ascorbic acid was generated based on selected concentrations. The stock solution of l‐ascorbic acid was prepared at a concentration of 1000 μg/mL using distilled water, which was used to make six serial dilutions of 500, 400, 300, 200, 100, and 50 μg/mL to achieve in‐well concentrations of 16.12, 12.90, 9.67, 6.45, 3.22, and 1.61 μg/mL. The measurement of the antioxidant effectiveness is presented as IC_50_ (the concentration required to scavenge 50% of the free radical). Results were obtained using the FluoStar Omega microplate reader, calculated using Equation (1), and presented as means ± standard deviation (SD).

### 2.6 Statistical analysis

Data were expressed as a mean ± SD, and the difference between the control and treated samples was determined using the Student *t*‐test, which was considered significant when *p* < .05. The IC_50_ values for the antioxidant effectiveness were calculated using the GraphPad Prism 5®. For the cytotoxicity studies, the IC_50_ values were determined by converting the PPEs concentrations into their corresponding logarithmic values and the nonlinear inhibitor regression equation (log [inhibitor] vs. normalized response–variable slope equation) was selected. An automatic outlier removal was selected.

## 3 RESULTS

### 3.1 Measurements of pH

**FIGURE 3:**
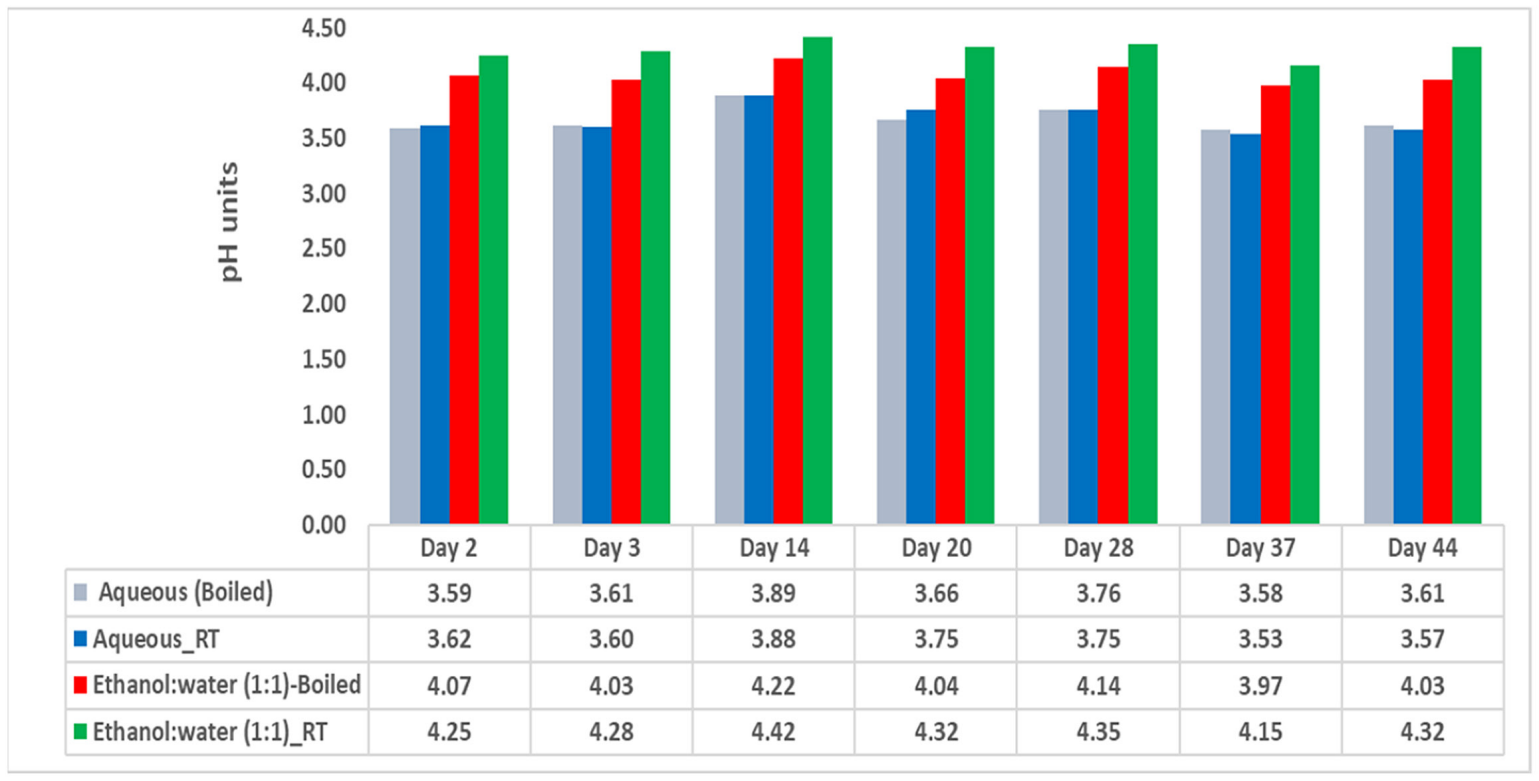
The pH values for the pomegranate peel aqueous and ethanol:water (1:1) extracts.

The pH was measured for the four water‐containing extracts. In general, the ethanol:water extracts showed relatively higher pH values (average 4.19) compared to the corresponding aqueous extracts (average 3.67). Boiling was noted to decrease the pH of the extracts, though the overall pH values remained consistently acidic with a maximum change of 1.5% over a period of 6 weeks (Figure 3).

### 3.2 Antibacterial activity of pomegranate peel extracts

The crude of each PPE was evaluated for its antimicrobial effectiveness at the highest concentration (40 mg/mL). This concentration had a remarkable inhibition on the growth of all the bacteria studied.

#### 3.2.1 Antimicrobial activity against Gram‐negative foodborne bacteria

**FIGURE 4:**
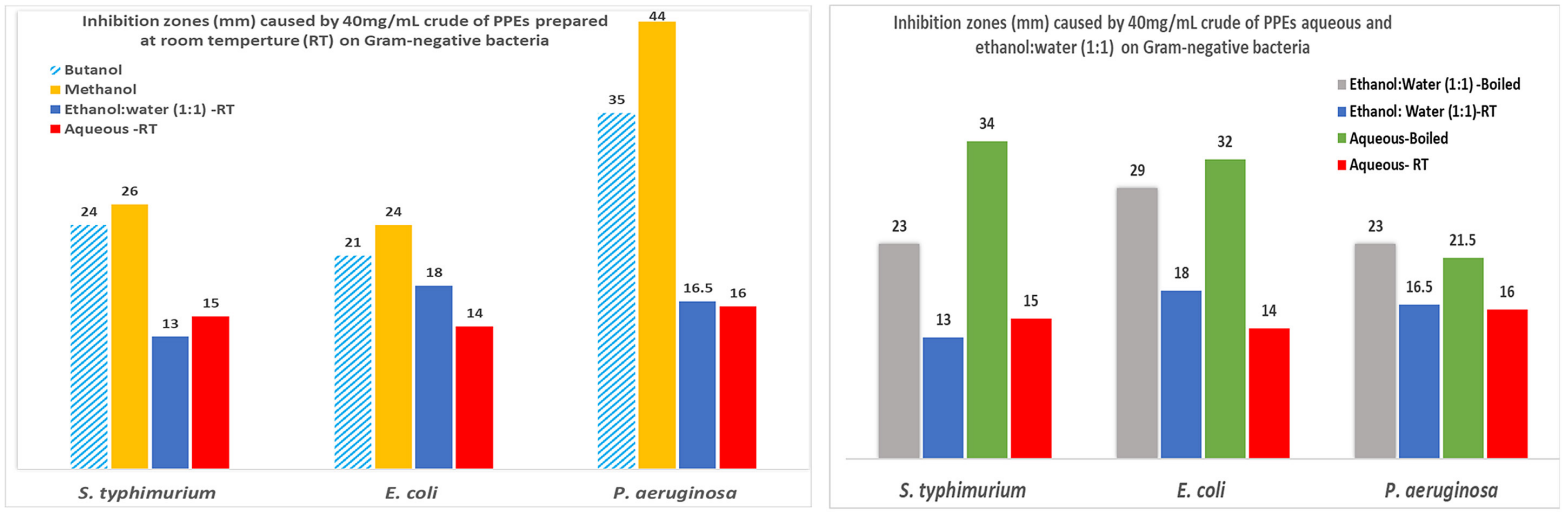
The antimicrobial activity of 40 mg/mL crude of pomegranate peel extracts (PPEs) against selective Gram‐negative bacteria.

Figure 4 demonstrates that the crudes of PPEs prepared at room temperature (RT) inhibited the growth of the Gram‐negative bacteria. The trend of inhibition caused by 40 mg/mL of each crude was averagely the highest by methanol, followed by butanol, ethanol:water (1:1), and the aqueous extract. It is noticeable that boiling increased the inhibitory effect of the water‐containing PPEs over the three bacteria studied, showing a trend of ethanol:water (boiled) > ethanol:water (RT) and aqueous (boiled) > aqueous (RT).

#### 3.2.2 Antimicrobial activity against Gram‐positive bacteria

This study evaluated the antimicrobial activity of the crude for each of the six PPEs on selected nonspore and spore‐forming Gram‐positive bacteria using the highest crude concentration of 40 mg/mL.

##### Antimicrobial activity against Gram‐positive nonspore‐forming bacteria

**FIGURE 5:**
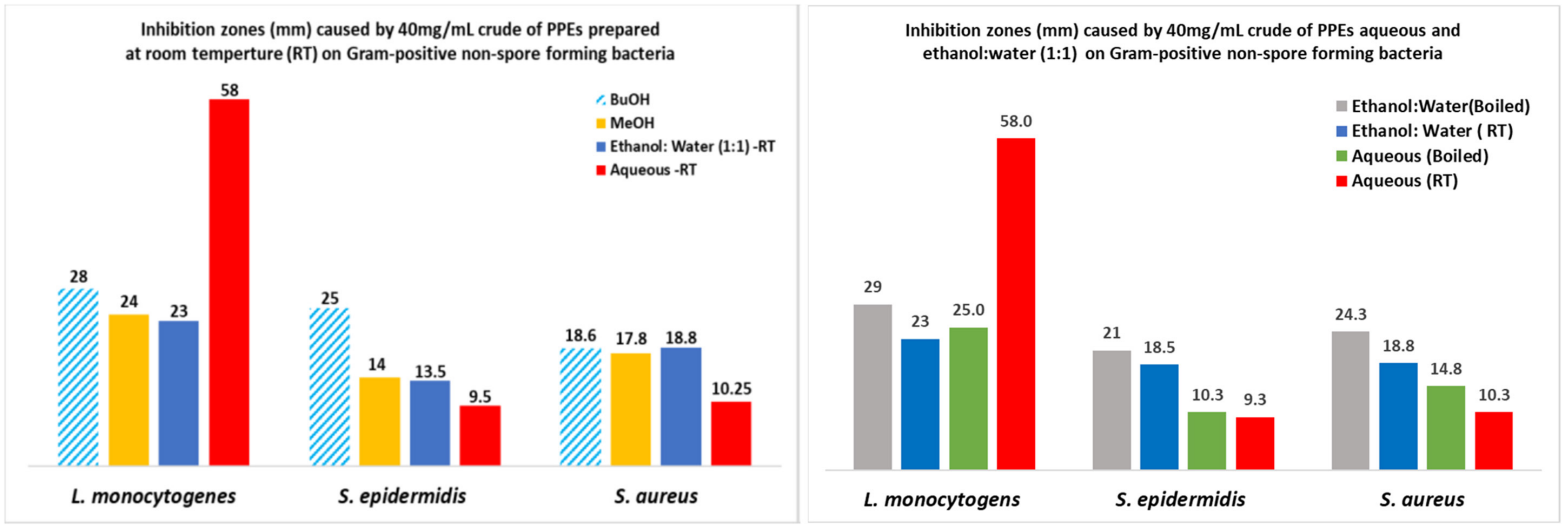
The antimicrobial activity of 40 mg/mL crude of pomegranate peel extracts (PPEs) against Gram‐positive nonspore‐forming bacteria.

The crude of PPEs of methanol exhibited a relatively higher inhibition (19–28 mm) compared to those of the butanol and ethanol:water (RT), which exhibited an inhibition in a moderately narrow range of (14–24 mm) and (18.5–23 mm), respectively. The presence of organic solvent appears to have inhibitory effects on the Gram‐positive nonspore‐forming bacteria except for *L. monocytogenes*, which was remarkably inhibited by the 100% water. Figure 5 shows that the aqueous extract prepared at room temperature (RT) had the highest inhibition against *L. monocytogenes* (58 mm) compared to all the other solvent systems. Interestingly, boiling decreased the inhibition of the aqueous extract by 50%, making its inhibitory effect almost equivalent to those of the methanol and ethanol:water (RT). However, boiling increased the inhibition of the ethanol:water on all bacteria, causing a higher inhibition than that caused by the ethanol:water (RT).

Overall, the PPEs had the most effective inhibition against *L. monocytogenes* and the least inhibition against *S. epidermidis*.

##### Antimicrobial activity against Gram‐positive spore‐forming bacteria

**FIGURE 6:**
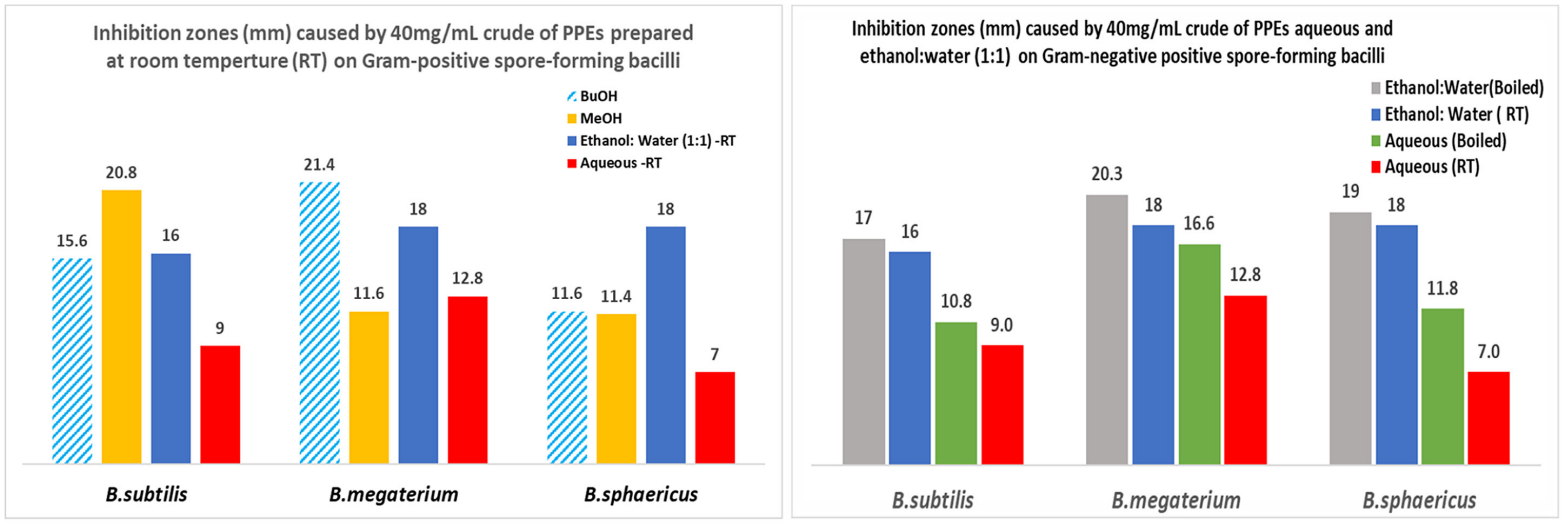
The antimicrobial activity of 40 mg/mL pomegranate peel extracts (PPEs) on selective Gram‐positive spore‐forming *Bacilli*.

The performance of crude of both the methanol and butanol PPEs was inhibiting the growth of the three *Bacillus* species in the range of (12–21 mm). The highest inhibition (~21 mm) was observed with methanol on *B. subtilis* and butanol on *B. megaterium*, while the highest inhibition on *B. sphaericus* was recorded with ethanol:water (RT) at 18 mm. The aqueous extracts caused the least inhibition (7–13 mm), which was enhanced by the effect of ethanol in the ethanol:water extract, which was consistent across the *Bacillus* species. Figure 6 illustrates the boiling effect on the aqueous and the ethanol:water PPEs as these extracts demonstrated an increased inhibition of the bacterial growth compared to the inhibition exhibited with their counterparts prepared at room temperature showing an inhibition trend as ethanol:water (boiled) > ethanol:water (RT) > aqueous (boiled) > aqueous (RT).

#### 3.2.3 Antibiotics susceptibility test

**FIGURE 7:**
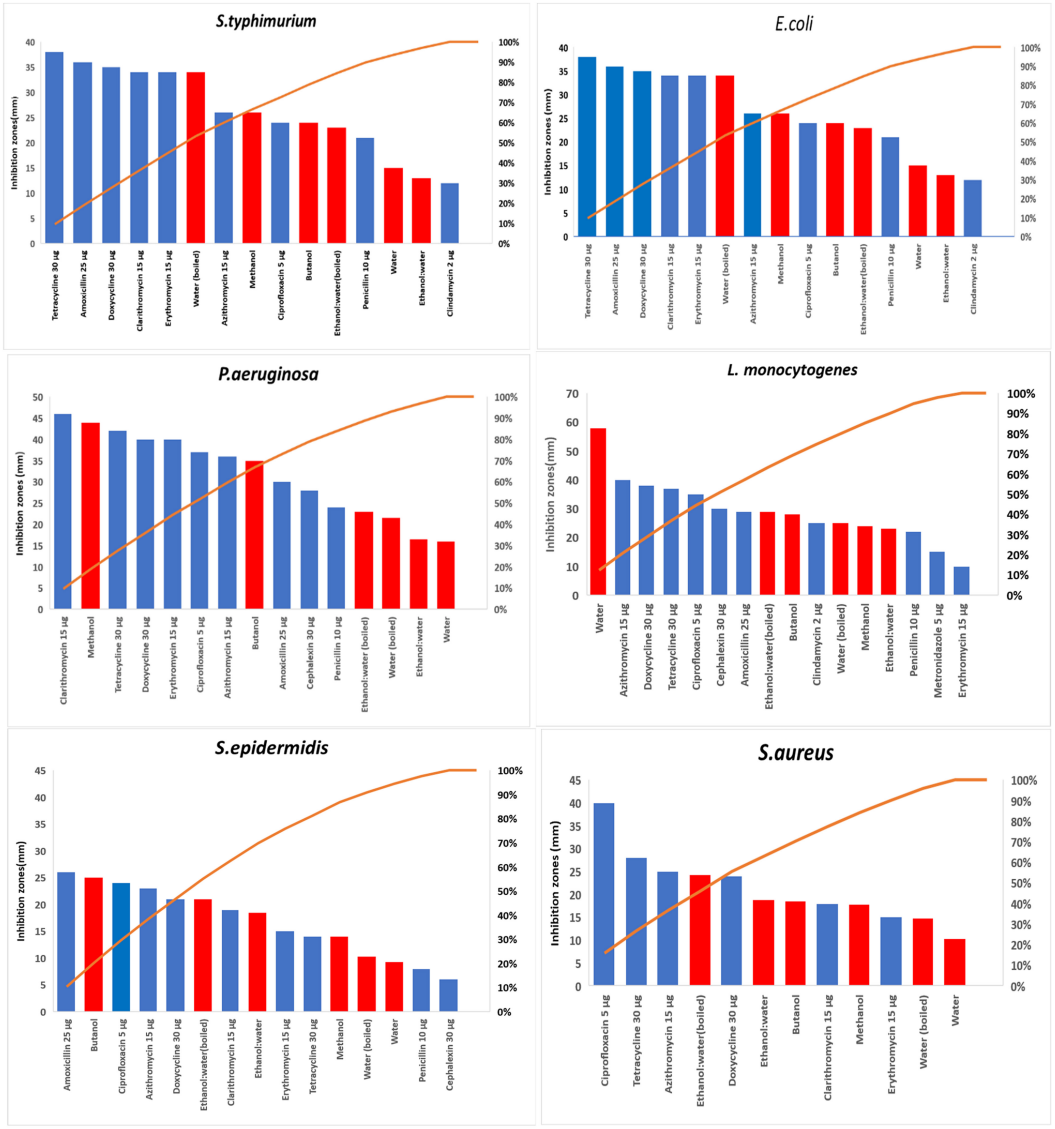
The performance index of the tested antibiotics and pomegranate peel extracts (PPEs) on the American Type Culture Collection (ATCC) bacteria.

Table 2 summarizes the inhibition caused by 40 mg/mL crude of all PPEs compared to the inhibition caused by standard antibiotics on the tested ATCC bacteria. It provides a useful guide to generate a performance index showing the inhibitory effectiveness of PPEs compared to that exhibited by the tested antibiotics on the same bacterium (Figure 7). For example, the performance index of the boiled aqueous PPE against *S. typhimurium* and *E. coli* was similar in antimicrobial efficacy to five out of nine antibiotics (~55%) (Erythromycin, Tetracycline, Doxycycline, Amoxicillin, Clarithromycin). It was noted that the water‐containing extracts (RT and boiled) had the least inhibitory effect on Gram‐positive nonspore‐forming bacteria, with the exception of the aqueous crude (RT) on *L. monocytogenes*, which outperformed all 10 antibiotics.

**TABLE 2:** The inhibition (mm) caused by 40 mg/mL crude of all pomegranate peel extracts (PPEs) and antibiotics on the American Type Culture Collection (ATCC) bacterial strains.

	*S. typhimurium*	*E. coli*	*P. aeruginosa*	*L. monocytogenes*	*S. epidermidis*	*S. aureus*
Water (RT)	15.0	14.0	16.0	58.0	9.3	10.3
Water (boiled)	34.0	32.0	34.0	25.0	10.3	14.8
Methanol	26.0	24.0	44.0	24.0	14	17.8
Butanol	24.0	21.0	35.0	28.0	25.0	18.5
Ethanol:water (RT)	13.0	18.0	16.5	23.0	18.5	18.8
Ethanol:water (boiled)	23.0	29.0	21.5	29.0	21.0	24.3
Antibiotics						
Clarithromycin 15 μg	34	17	46	0	19	18
Metronidazole 5 μg	0	0	0	15	0	0
Lincomycin 2 μg	0	0	0	0	0	0
Cephalexin 30 μg	0	16	28	30	6	0
Amoxicillin 25 μg	36	22	30	29	26	0
Doxycycline 30 μg	35	12	40	38	21	24
Ciprofloxacin 5 μg	24	23	37	35	24	40
Penicillin 10 μg	21	8	24	22	8	0
Azithromycin 15 μg	26	23	36	40	23	25
Clindamycin 2 μg	12	6	0	25	0	0
Tetracycline 30 μg	38	11	42	37	14	28
Erythromycin 15 μg	34	13	40	10	15	15

Abbreviation: RT, room temperature.

#### 3.2.4 Minimum inhibitory concentration (MIC)

**FIGURE 8:**
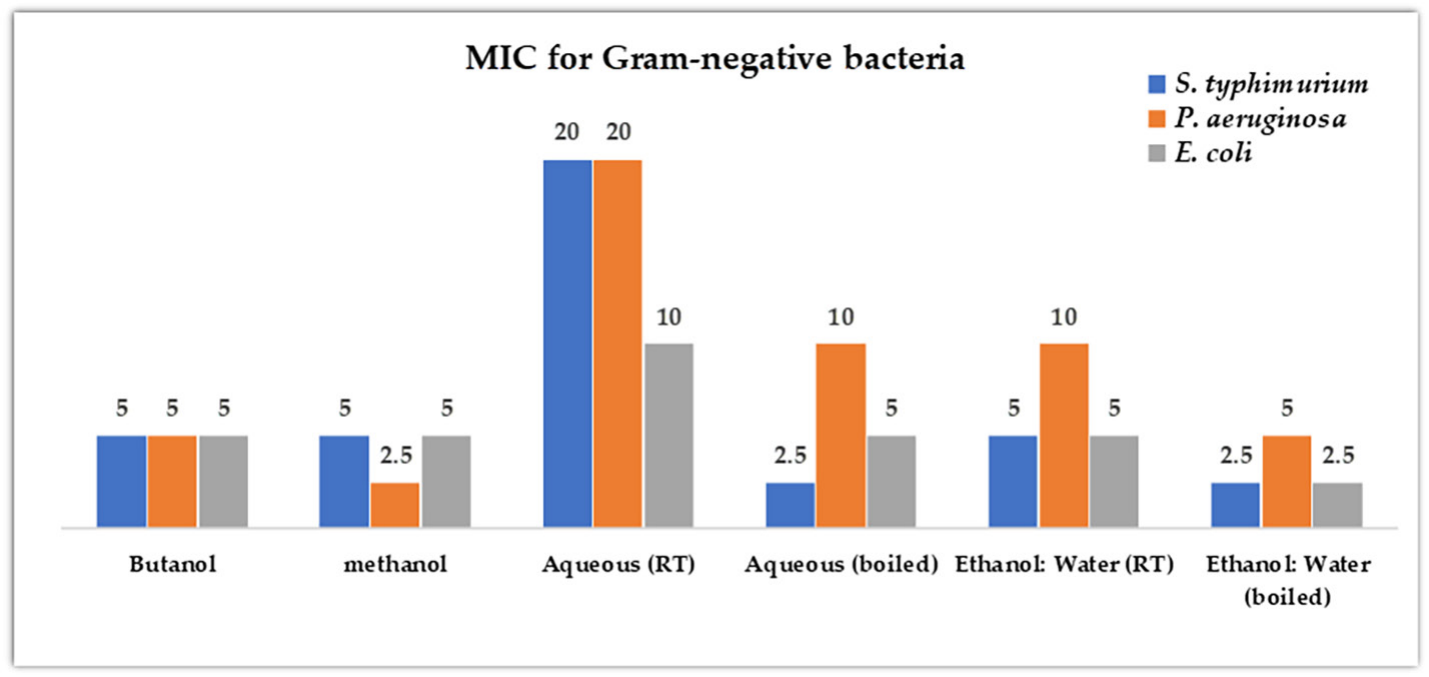
The minimum inhibitory concentrations (MICs) (μg/mL) of the crude of pomegranate peel extracts (PPEs) on the Gram‐negative American Type Culture Collection (ATCC) bacteria. RT, room temperature.

From the 40 mg/mL crude stocks, five dilutions (20, 10, 5, 2.5, and 1.25 mg/mL) of each extract were prepared and evaluated only against the ATCC Gram‐negative foodborne bacteria (*S. typhimurium*, *E. coli*, *P. aeruginosa*). Figure 8 shows that the MIC values of the pure organic solvents (butanol and methanol) did not exceed 5 mg/mL and highest MIC values observed were of those induced by the water‐containing extracts. It is also noted that boiling decreased the MIC values for the aqueous and the ethanol:water PPEs.

### 3.3 Cytotoxicity in vitro screening using MTT assay

Prostate cancer cells (PC3), ovarian cancer cells (SKOV‐3), and colon cancer cells (HCT116) were selected for assessing their proliferation when treated with the six PPEs. Pursuant to the International Organization for Standardization (ISO 10993‐5), the percentages of cell viability above 80% are considered noncytotoxic, within 80%–60% weak, 60%–40% moderate, and below 40% strongly cytotoxic, respectively (López‐García et al., 2014). The IC_50_ values for the crudes of the PPEs on all cells are summarized in Table 3.

**TABLE 3:** The half maximal inhibitory concentration (IC_50_) (μg/mL) values for the crudes of the pomegranate peel extracts (PPEs) on the examined cells.

	PC3	HCT116	SKOV‐3	Fibroblast
Methanol	<0.1	25.24	Not toxic	Not toxic
Aqueous (Room temp.)	<0.1	27.57	Not toxic	Not toxic
Aqueous (Boiling)	0.805	21.45	Not toxic	Not toxic
Ethanol:water (Boiling)	14.32	29.69	Not toxic	Not toxic
Ethanol:water (Room temp.)	20.08	37.25	Not toxic	Not toxic
Butanol	22.084	42.55	Not toxic	Not toxic

**FIGURE 9:**
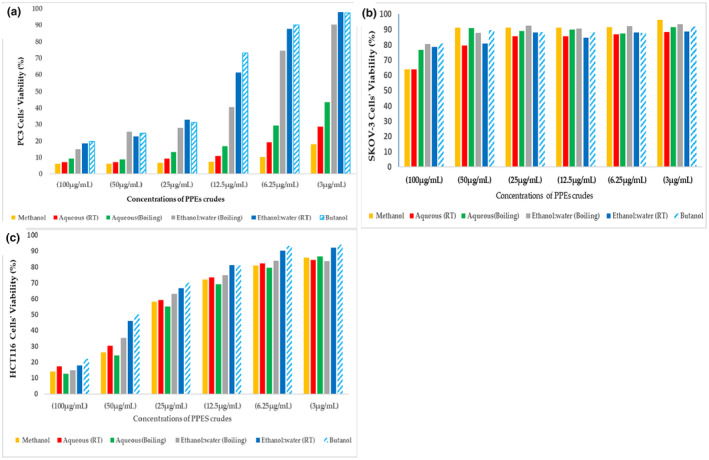
The inhibitory effect of pomegranate peel extracts (PPEs) on the proliferation of (a) prostate cancer cells (PC3), (b) ovarian cancer cells (SKOV‐3), and (c) colon cancer cells (HCT116).

The cellular proliferation was impacted differently per the cancer cell lines (Figure 9). Overall, of the two lines of cancerous reproductive cells, PC3 cells were the most responsive to the antiproliferative effects of the six PPEs (Figure 9a) compared to SKOV‐3 cells (Figure 9b) which were minimally affected even at the highest concentrations (100 μg/mL). With respect to PC3, all the PPEs showed a decrease in toxicity as the concentrations increased. All extracts above a concentration of 25 μg/mL exhibited noticeable toxicity, however, the methanol and aqueous (RT) were consistently the most toxic (below 40%) against the proliferation of PC3 cells at all tested concentrations (Figure 9a). While the HCT116 cells followed the PC3 pattern of decreasing viability as concentrations increase, there was not any significant toxicity until the concentration of 50 μg/mL and above (Figure 9c). It was also observed that the proliferation of fibroblast cells was not significantly impacted by all the PPEs, as the IC_50_ values were determined to be nontoxic.

### 3.4 Antioxidant activity using l‐ascorbic acid

**FIGURE 10:**
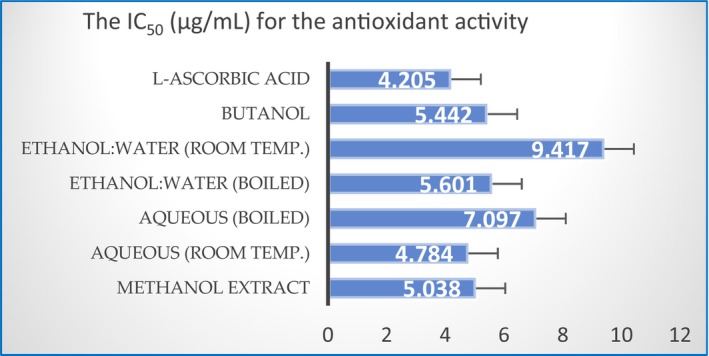
The half maximal inhibitory concentration (IC_50_) for the antioxidant activity of the six pomegranate peel extracts (PPEs). The bars represent the standard deviation.

The protocol for measuring the antioxidant activity was outlined in Sections 2.5 and 2.6. Figure 10 shows that values of the IC_50_ of all PPEs were higher than those of the ascorbic acid with a trend as ethanol:water (RT) > aqueous (boiled) > ethanol:water (boiled) > butanol) > methanol) > aqueous (RT). The IC_50_ value for the ethanol:water mixture (RT) was almost twice that of the ascorbic acid. It is noted that boiling decreased the IC_50_ of the ethanol:water extract (RT), contrary to it causing an increased IC_50_ for the aqueous extract, indicating an enhancement of its antioxidant activity.

## 4 DISCUSSION

Pomegranate has attracted the attention of researchers for its potential health benefits. This fruit has been reported to have an abundance of bioactive compounds that possess anti‐inflammatory, antibacterial, and antitumor properties. However, studies reporting the biological effectiveness of PPEs follow different protocols for preparation of these extracts. This poses a challenge in determining the PPEs with the best performance for diverse purposes, and thus a challenge in developing quantitative assessments for the possible benefits of pomegranate peel. This study compares the antimicrobial, cytotoxic, and antioxidant activities of pomegranate peel using six different PPEs prepared with the same method for extraction. The four water‐containing PPEs were acidic, even though boiling caused a slight decrease in the pH of the aqueous extract, but did not affect the pH of the ethanol:water mixture. These findings are in agreement with those of previous research, which had reported a low pH of pomegranate juice (ranging from 2.9 to 3.75) which was attributed to its high citric acid content, while the acidity of ethanol:water (1:1) and methanol PPEs was attributed to the highest content of some phenols, especially punicalagins (Kandylis & Kokkinomagoulos, 2020; Kokkinomagoulos et al., 2020; Malviya et al., 2014; Tamborlin et al., 2020).

Our study assessed the antimicrobial effect of the PPEs on Gram‐negative and Gram‐positive bacteria that are associated with food spoilage. The results showed that the PPEs differ in their inhibitory effectiveness on the growth of these bacteria. In general, two main factors contributing to this variation would be the bacterial structure and the extracted bioactive compounds.

With respect to Gram‐negative bacteria, among the PPEs prepared at room temperature, the extracts of the pure organic solvents had a stronger bacterial inhibition relative to that of the water‐containing extracts showing a trend as methanol > butanol > ethanol:water (RT) > water (RT). Boiling enhanced the effectiveness of the inhibitory effects for the four water‐containing extracts and the boiled ethanol:water mixture had the most effective inhibition on *S. typhimurium* and *E. coli*. The disruption of the outer membrane, which consists of lipopolysaccharide, is most likely responsible for the growth inhibition of the Gram‐negative bacteria (Breijyeh et al., 2020).

With respect to the Gram‐positive bacteria, both spore‐ and nonspore forming, the six PPEs inhibited the bacterial growth, and boiling impacted this inhibition. With the exception of *L. monocytogenes*, boiling increased the inhibitory effect of the aqueous and ethanol:water extracts showing a trend of ethanol:water (boiled) > ethanol:water (RT) > aqueous (boiled) > aqueous (RT). Against *Listeria*, however, the crude of the room temperature aqueous PPE had the highest inhibitory effect among the six PPEs and the 10 antibiotics tested. *Listeria* is a unique pathogenic bacterium as it adheres to various surfaces and can multiply at 4°C which is the refrigeration temperature (Meloni, 2014). It is referred to as a psychrophilic bacterium, meaning that it has certain cell membrane adaptations including those given as follows: increased polyunsaturated to saturated fatty acid ratios in membrane phospholipids, changes lipid class composition, reduces the size and charge of lipid head groups, which affects the packing of phospholipids, and facilitates the conversion of trans‐ to cis‐isomeric fatty acids (Gounot, 1986). *L. monocytogenes* was reported to be generally sensitive to polyphenols, compared to other bacteria, as some of these compounds can interact with the sulfhydryl groups in the *Listeria* cell membrane inhibiting selective membranous enzymes (Bouarab‐Chibane et al., 2019; Singh et al., 2018). Several studies were conducted to determine the factors affecting the extraction of bioactive compounds from the pomegranate peel, thus impacting their yield and stability. In addition to temperature, other factors can affect the yield of bioactive compounds during the extraction process, including the solvent, extraction method, and time of extraction. The polarity of the solvents used in this study for the extraction determined the categories and yield of the bioactive compounds in the PPEs. It was reported that the optimal conditions for obtaining the highest yield of polyphenolic, gallic acid, and total flavonoid content from pomegranate peel were a solid‐to‐solvent ratio of 1:30, a temperature of 50°C, and a 45‐min extraction time (Sood & Gupta, 2015). It was highlighted that the content of punicalagin, a hydrolysable tannin in pomegranate peel, decreased up to 70.05% as the temperature increased to 40°C under neutral pH (Rakshit & Srivastav, 2022), and the hydrolysable tannins were more stable in methanol or ethanol solutions than in aqueous solutions at temperatures ranging from 70°C to 100°C (Wang, Alseekh, et al., 2019; Wang, Chen, et al., 2019). Additionally, the methanolic extracts were reported to contain a significant yield of antioxidants compared to their yield in the ethanol and ethyl acetate extracts (Arab et al., 2011). The study of Muñiz‐Márquez et al. (2021) reported that the most effective extraction conditions for bioactive extracts from pomegranate peel was by using ethanol:water mixture at 93.6°C. The antibacterial effect of the aqueous PPE was suggested to be due to its significant content of chlorogenic acid, a phenolic compound that has the ability to interact with the bacterial outer membrane, which ruptures the cell membrane and depletes the intracellular components leading to bacterial death (Borges et al., 2013; Cai et al., 2019). A scanning electron microscopy study confirmed that some plant extracts had a disruptive effect on the cell membrane of *L. monocytogenes*, triggering flagella damage and loss (Ceruso et al., 2020). An in vivo trial for antibacterial activity confirmed that the PPE significantly reduced the bacterial load of *L. monocytogenes* on fresh‐cut apple, pear, and melon and maintained a low number of bacterial cells throughout the storage period (Belgacem et al., 2020).

This study selected solvents of variable polarities, with water being the most polar and butanol being the least polar. The trend of polarity is water (1) > methanol (0.76) > ethanol:water mix (0.71) > butanol (0.39). Studies used different solvents for preparing plant extracts, including organic solvents such as methanol, ethanol, acetone, ethyl acetate, and their combinations. The methanolic extracts are popular and have been recognized for inhibiting the growth of several Gram‐positive and Gram‐negative bacteria. Their strong antimicrobial effect was attributed to their higher content of phenols and flavonoids than other extracts (El‐Hadary & Taha, 2020; Saparbekova et al., 2023). Our results showed clearly that the methanol PPE was more effective than butanol against the Gram‐negative bacteria, and only on *B. subtilis* among all the Gram‐positive bacteria. The difference in the effect of methanol on Gram‐positive and Gram‐negative bacteria is probably due to the effect of other factors such as: the interaction of the bioactive compounds with the bacterial membrane, nature of the extracted compounds, and their yields.

The aqueous extracts of different plants usually yield significantly higher amounts of antioxidants compared to the ethanolic extracts of the same plants because of the high polarity of water (Bouarab‐Chibane et al., 2019; Kokkinomagoulos et al., 2020). The water‐soluble phenols are recognized as powerful antibacterial compounds which may act synergistically as mixtures to enhance the antimicrobial effects of water‐containing PPEs. Among these compounds are ellagitannins, a phenol with central glucose units, anthocyanins (pelargonidin‐3‐galactose and cyanidin‐3‐glucose) and flavonols (quercetin and myricetin) (Melgarejo‐Sánchez et al., 2021; Mo et al., 2022; Pirzadeh et al., 2021). Many polyphenolic compounds can modify the bacterial cell membrane permeability (Bouarab‐Chibane et al., 2019; Fei et al., 2019), while others such as flavonoids can form complexes with dissolved proteins that are located outside the bacterial cell that may inhibit the DNA synthesis (Bouarab‐Chibane et al., 2019, Cushnie and Cushnie & Lamb, 2011). Tabaraki et al. (2012) reported that using a solvent mixture for ultrasound‐assisted extraction of polyphenols resulted in their highest yield compared to using only water or ethanol.

The evaluation of PPEs for their antiproliferative properties showed that the methanol inhibited the proliferation of the PC3 and SKOV‐3 cells (below 40%) the most, followed by the aqueous extract prepared at room temperature. The antiproliferative effect of all the PPEs on PC3 and HCT116 was reflected in a concentration‐dependent trend with PC3 cells displaying greater sensitivity, while the SKOV‐3 proliferation remained comparable to the control cells. Studies reported that the cell viability was reduced below 40% by different concentrations of methanolic PPE, and low doses exerted potent toxicity on the tested cancer cells. It was suggested that the significant antiproliferative properties of different PPEs were due to their high content of phytochemicals reputed for anticancerous activity that lead to their use in carcinopreventive adjuvant therapies (Choudhari et al., 2020). Such components were reported to affect the transcriptional factors, pro‐inflammatory mediators, anti‐apoptotic proteins, cell cycle regulator molecules, and protein kinases in various cell lines. The majority of these active compounds are polyphenols, such as ellagic acid and punicalagin, which were isolated in a significant quantity from different PPEs (Wang & Martins‐Green, 2014). Some of the polyphenols which induced apoptosis might also hinder angiogenesis and metastasis in various malignant cells through several signaling pathways, such as interactions with several cancer‐related proteins, P53, and WNT, which play a vital role in the progression of many tumors (Cheshomi et al., 2021; Jia et al., 2019). Another study reported that a PPE‐treated hepatic cancer cell (HepG2) revealed necrosis and caused cell membranes and intranuclear eosinophilic structures with nuclear membranes to burst. Under the effect of 80% ethanol extract, an upregulation in the expression of pro‐apoptotic gene and downregulation in the expression of anti‐apoptotic gene were substantially expressed (Nasr et al., 2023). In a study of Parameswari et al. (2019), results revealed the antiproliferative effect of aqueous:ethanol extract (1:1) on breast cancer (MCF7) and colon cancer cells (HCT116), but to a lower extent on the normal fibroblasts. Different reports indicated that methanol–water PPEs selectively had antiproliferative effect on lung and prostate cancer cells, with insignificant toxicity to ovarian cancer (SKOV‐3) cells (Keta et al., 2020; Park et al., 2016; Portillo‐Torres et al., 2019). This study showed that the PPEs were not toxic to fibroblasts treated with all the tested concentrations of PPEs. Multiple studies reported that the tropical application of PPE induced the wound healing via promotion of collagen production and stimulation of several growth factors (Singh et al., 2023). Further clinical studies can be helpful to assess the potential of different PPEs to heal wounds.

The bioactive compounds possess anticancer activity often by decreasing the free radicals and oxidative stress generated in cells, and thus are considered antioxidants (Greenwell & Rahman, 2015). Several PPEs were reported for their potently scavenged DPPH radicals due to their content of alpha‐glucosidase in the following order: ethyl acetate > methanol > hexane. The detection of hydroxymethylfurfural and 4‐fluorobenzyl alcohol compounds might play a role in antioxidant and antimicrobial activities of the PPEs (Barathikannan et al., 2016; Belgacem et al., 2021). This study revealed that the value of IC_50_ of the aqueous extract (RT) was the closest to that of the ascorbic acid, indicating its highest antioxidant potency among the six tested PPEs. However, the IC_50_ of the ethanol:water mixture (RT) was the highest, indicating the lowest antioxidant activity. According to several studies, the antioxidant activity of pomegranate peel is attributed to the total phenolic compounds with their recovery linked to several factors including the cultivation region, extraction method, and the most appropriate extraction solvent used. The polarity of solvents influences the extraction efficiency of phenolic compounds, and thus dictates the biological activities exhibited by the plant extracts.

Table 4 shows the correlation between the polarity trend and the IC_50_ of the antioxidant activity for the solvent systems used in this study. With the exception of butanol, the table shows that when the polarity of the extraction solvent increased, the exhibited IC_50_ of the extract decreased demonstrating a higher antioxidant effectiveness.

**TABLE 4:** The polarity trend and the antioxidant activity at half maximal inhibitory concentration (IC_50_) of the solvent systems.

PPEs	IC_50_	Polarity
Aqueous	4.784	1
Methanol	5.038	0.76
Ethanol:aqueous (1:1)	9.417	0.71
Butanol	5.442	0.39

Studies indicated that the less polar solvents often extract smaller amounts of phenolic compounds, and therefore, these extracts possess a lesser potential for scavenging free radicals (Derakhshan et al., 2018; Saparbekova et al., 2023), which may be attributed to the slightly lower value of IC_50_ of butanol PPEs compared to that of the methanol PPE indicating its higher antioxidant activity.

It is obvious that boiling had an effect on the stability of the compounds extracted in the water‐containing PPEs. The study showed that boiling increased the antioxidant activity of the ethanol:water mixture, as indicated by lowering the IC_50_ value from 9.417 to 5.602. However, the effect of boiling on the aqueous extract was the opposite, as it increased the IC_50_ value from 4.874 to 7.097, indicating a decrease in the antioxidant effectiveness of the boiled aqueous extract. This might be due to degradation of some active phenols due to the high temperature during the extraction process.

In addition to the polarity of solvents, the solubility of phenolic compounds in different PPEs is governed by a complex interplay of parameters. A study conducted by Konsoula (2016) using methanol and ethanol solvents found that methanolic PPE had a better antioxidant index and the highest polyphenolic content, compared to the ethanolic extract. As methanol contains a smaller and more flexible aliphatic fragment compared to ethanol and butanol, thus it is easier to surround phenols with substituted carbons inside their aromatic ring. This might explain that methanol had a higher antioxidant activity than butanol, and also that it was more effective as an antimicrobial agent against some bacteria examined in this study. Further thorough investigation is suggested to determine the eco‐friendly conditions and the best methodology to use pomegranate peel for innovative applications in the food industry and for therapeutic uses.

## 5 CONCLUSION

The crudes of six PPEs at 40 mg/mL exhibited different levels of antimicrobial activities against the bacteria selected in this study. The highest effectiveness on inhibiting the growth of *L. monocytogenes* was caused by the aqueous extract prepared at room temperature, while the boiled aqueous extract had the highest inhibitory effect on *E. coli* and *S. typhimurium*. However, the boiled ethanol:water (1:1) caused the most effective inhibition against *S. aureus*, *B. sphaericus*, the second most effective inhibition against *S. epidermidis*, and a moderate effect on *B. megaterium*. The enhanced effectiveness with boiling, and the stability in pH over a period of time, leverage the use of pomegranate peel in applications needing high temperatures as well. It is recommended to have further studies using higher concentrations of the PPEs and their crudes. The aqueous extracts also have the potential to be used as an antiproliferative reagent against prostate cancer cells and possibly against colon cancer cells at high concentrations. It is suggested to evaluate the effect of PPEs on additional cells and to further investigate the mechanism of action. Overall, the findings clearly show that pomegranate peel possessed the potential to be an eco‐friendly novel source for natural compounds that can be implemented in the food industry as a natural antibacterial and food additive to prevent food spoilage and foodborne illnesses.

## AUTHOR CONTRIBUTIONS

**Lubna F. Abu‐Niaaj:** Conceptualization (equal); data curation (equal); formal analysis (equal); funding acquisition (equal); investigation (equal); methodology (equal); project administration (equal); resources (equal); supervision (equal); validation (equal); visualization (equal); writing – original draft (equal); writing – review and editing (equal). **Hala I. Al‐Daghistani:** Conceptualization (equal); data curation (equal); formal analysis (equal); funding acquisition (equal); investigation (equal); methodology (equal); project administration (equal); resources (equal); supervision (equal); validation (equal); visualization (equal); writing – original draft (equal); writing – review and editing (equal). **Ibrahim Katampe:** Conceptualization (equal); data curation (equal); formal analysis (equal); funding acquisition (equal); methodology (equal); validation (equal); visualization (equal); writing – original draft (equal); writing – review and editing (equal). **Bashaer Abu‐Irmaileh:** Data curation (equal); investigation (equal); methodology (equal); software (equal). **Yasser K. Bustanji:** Conceptualization (equal); data curation (equal); formal analysis (equal); funding acquisition (equal); investigation (equal); methodology (equal); software (equal); validation (equal); visualization (equal); writing – original draft (supporting).

## CONFLICT OF INTEREST STATEMENT

The authors declare there is no conflict of interest and that the research was conducted in the absence of any commercial or financial relationships that could be construed as a potential conflict of interest.

## Data Availability

The data that support the findings of this study are available from the corresponding author upon reasonable request.
